# Mirtazapine may show anti-hyperglycemic effect by decreasing GLUT2 through leptin and galanin expressions in the liver of type 1 diabetic rats

**DOI:** 10.22038/ijbms.2019.34529.8190

**Published:** 2019-06

**Authors:** Ezgi Bektur, Erhan Sahin, Cengiz Baycu

**Affiliations:** 1 Eskisehir Osmangazi University, Faculty of Medicine, Department of Histology and Embryology, Eskisehir, Turkey; 2 Okan University, Faculty of Medicine, Department of Histology and Embryology, Istanbul, Turkey

**Keywords:** Galanin, GLUT2, Leptin, Liver, Mirtazapine, Type 1 diabetes mellitus

## Abstract

**Objective(s):**

The aim of this study was to explore the molecular mechanism of mirtazapine with respect to energy metabolism in Streptozotocin-induced diabetic liver of rats by immunohistochemistry and Western blot.

**Materials and Methods:**

Twenty-one male Sprague-Dawley rats were assigned into 3 groups including control, type 1 diabetes mellitus (T1DM) group (55 mg/kg Streptozocin, IP) and T1DM+mirtazapine (20 mg/kg,PO) group. At the end of the experiment, blood glucose levels were measured and liver tissues were stained by Periodic acid–Schiff. Moreover, leptin and glucose transporter 2 (GLUT2) proteins were analyzed by western blot and immunohistochemistry; however, galanin were analyzed only by immunohistochemistry.

**Results:**

At the end of the study, in diabetes group, blood glucose level, GLUT2 and galanin expressions increased, while leptin expression decreased when compared to control group. Mirtazapine treatment restored the decreased leptin expression, and the increased blood glucose level and galanine expression to the level of the control group. It also decreased the GLUT2 expression even below the control group.

**Conclusion:**

We concluded that mirtazapine may show its anti-hyperglycemic effect by decreasing GLUT2 through altering the leptin and galanin expression in the liver of type 1 diabetic rats. Mirtazapine can be used as an antidepressant for T1DM patients and as a drug to reduce blood glucose level in T1DM.

## Introduction

Insulin, produced in the pancreatic β-cells, regulates glucose homeostasis by promoting glucose uptake and glycogen storage in the skeletal muscle, liver and fat cells. Type 1 diabetes mellitus (T1DM) is an autoimmune disease, and due to the gradual destruction of β-cells by T-cell, T1DM is characterized by deficiency or insufficiency of insulin in peripheral targets, hyperglycemia, metabolic and neural diseases, and a shorter life span (1). While the number of diabetic individuals in the world in 2013 was 382 millions, this number is thought to be 592 million in 2035 (2).

Significantly decreased levels of insulin and elevated blood glucose (hyperglycemia) are observed in the plasma of diabetic rats. As a result of hyperglycemia, reactive oxigenic radicals are produced due to various pathways such as increased glycation products, activation of protein kinase C, overproduction of mitochondrial superoxides and degradation of redox balance. As a result, many different systems of the body are affected and over time can lead to serious complications. These include diabetic neuropathy, nephropathy, disease of cardiovascular system, and macrovascular complications including liver and peripheral vascular diseases.

Metabolically, the liver, as a complex organ, plays a role in the metabolism and storage of lipids. The efficacy of carbohydrate metabolism plays an important role in blood glucose homeostasis. It provides a balance between the uptake and storage of glucose through glycogenesis. Insulin stimulates glycogenesis in the liver, but inhibits glycogenolysis. Imbalance in glucose regulation resulting from diabetes can lead to chronic tissue and organ damage (3).

Leptin, released from adipocytes, is a peptide hormone with molecular weight of 16 kDa. Leptin that is trasmitted to brain through the bloodstream, controls energy homeostasis by binding to its receptor in the hypothalamus. Leptin reduces nutrient uptake and increases energy consumption through the effect on the hypothalamus (4). Leptin also has a glucose-lowering effect by a mechanism independent of insulin in uncontrolled diabetes, and it normalizes the hepatic glucose production by increasing glucose uptake rate in peripheral tissues such as heart, brown adipose tissue and skeletal muscles. In addition, leptin signals released from the adipocytes are transmitted to the hypothalamus via the bloodstream and inhibit fat accumulation and food uptake (5). Low level leptin is associated with depression-like behavior in rodents. In many pharmacological studies on rodent models with depression-like behavior, it has been reported that leptin is regulated by leptin receptor activation in specific limbic regions, such as the hippocampus, and leptin has an antidepressant-like effect (4). In humans and in rodents, various leptin receptor isoforms are widely distributed in many organs, including the pancreas, liver, heart, kidney, adipose tissue, and brain (6). 

Galanin is a 29/30 amino acid peptide that was discovered in 1983 in the porcine intestine (7). This neuroendocrine peptide stimulates food intake and regulates energy metabolism (8, 9). These actions are mediated via three galanin receptor subtypes. GalR1–3 are widely distributed in the nervous system and pancreas as well as gut (10). Galanin has an important and complex function on glucose hemostasis. It inhibits glucose-stimulated insulin release in human and animal models. Galanin also has a significant role in elevation of insulin sensitivity to promote glucose clearance in skeletal muscle, heart muscle and adipose tissue, though glucose transporters (11). Fasting insulin levels can maximally stimulate brain cortical glucose metabolism in humans, and that the insulin-induced increase in glucose uptake may involve the recruitment of the glucose transporter 1 (GLUT1), GLUT2 and GLUT4 to the plasma membrane (12-15).

Living with diabetes can be stressful and can also cause symptoms of depression. Leptin, an anorexigenic hormone, is directly proportional to body fat. Therefore, weight gain with antidepressant therapy is associated with increased leptin levels (16). In addition, some antidepressants are used as pain relievers instead of agents such as pregabalin that is used clinically in diabetic neuropathy, which is one of the complications caused by diabetes.

Mirtazapine is an atypical antidepressant with noradrenergic and serotonergic activity. It is a selective antagonist of 5-hydroxytryptamine (5-HT2) and 5-HT3 serotonin receptors in the central and peripheral nervous system, while blocking α2 adrenergic auto and heteroreceptors that cause reducing the glucose concentration in the plasma without altering the insulin response. Mirtazapine increases the neurotransmission of serotonin via the 5-HT1 receptor. It blocks histaminergic (H1) and muscarinic receptors (17). Mianserin, an analogue of mirtazapine, increases insulin 1 (Ins1) mRNA levels, which are expressed at low levels in diabetic rats. From this, it has been shown that subacute administration of mianserin over a long-term in hyperglycemia model has a prominent anti-hyperglycemic effect (18).

When we take into account all of these factors, our aim is to investigate whether mirtazapine has a direct effect on glucose metabolism, leptin, galanin and GLUT2 expression on the diabetic liver.

## Materials and Methods

***Animals***

The treatment and care of animals were according to the guidelines for the study of experimental pain in conscious animals and were approved by the Animal Care Committee of the Eskisehir Osmangazi University. Male Sprague–Dawley rats (250–320 g) were housed three per cage under controlled illumination (12 hr light/dark cycle) and environmental conditions. 

***Streptozocin induction of diabetes***

Twenty-one male Sprague- Dawley rats were injected with Streptozocin (STZ; Sigma, St. Louis, MO) at 8 weeks of age to induce type 1 diabetes. After 72 hr from 55 mg/kg STZ injection, rats were fasted for 12 hr before blood glucose measurement (19). Solutions were made fresh immediately prior to injection and STZ was dissolved in ice cold 10 mM sodium citrate buffer with 0.9% NaCl at pH 4.5, and then filter sterilized. Control rats were injected with sodium citrate buffer only. Animals reached hyperglycemia (blood glucose >300 mg/dl) within 72 hr following initial.

***Blood glucose measurements***

Blood glucose measurement was performed both before starting experiment and at the last day of experiment. Rats were considered diabetic when blood glucose levels were ≥300 mg/dl.

***Drugs and drug administration***

From 4^th^ weeks after STZ injection to 6^th^ weeks, 20 mg/kg mirtazapine (Zestat 30 mg, Abdi İbrahim Pharmaceutical industry and trade joint-stock company, İstanbul) was applied by intragastric gavage tube every day. At the end of the study period, all groups received anesthesia with 100 mg/kg sodium thiopental. Liver tissues were isolated for western blot and immunohistochemistry.

***Tissue preparation for Periodic Acid Schiff (PAS) staining***

Rats received anesthesia with 100 mg / kg sodium thiopental, injected with 150 μl of heparin (BD Biosciences) into the ventricle, and then transcardially perfused with 10% formaldehyde in 1x phosphate-buffered saline (PBS, pH 7.4). Liver tissues were dissected, and postfixed in 10% formaldehyde for 24 hours. Following fixation, paraffin blocks were prepared by following the appropriate procedure to the received tissues. Serial sections of 4 μm thickness were prepared from each of paraffin blocks. One day after being kept at 37 ^°^C, the temperature of the oven was increased to 60 ^°^C at the next day and it was waited for 1 hr. After passing through the xylol and reduced alcohol fractions, sections were oxidized in 0.5% periodic acid solution for 5 min. Then, sections rinsed in distilled water and placed in Schiff reagent for 15 min (Sections became light pink color during this step). After we washed sections in lukewarm tap water for 5 min (Immediately sections turn dark pink color), counterstaining was performed in Mayer’s hematoxylin for 1 min. Then, we washed sections in tap water for 5 minutes and dehydrated and coversliped using a synthetic mounting medium.

***Tissue preparation for immunohistochemistry***

Liver tissues were dissected, and postfixed in 10% formaldehyde for 24 hr. Following fixation, paraffin blocks were prepared by following the appropriate procedure to the received tissues. Serial sections of 4 μm thickness were prepared from each of paraffin blocks. One day after being kept at 37 ^°^C, the temperature of the oven was increased to 60 ^°^C the next day and it was waited for 1 hr. After passing through the xylol and reduced alcohol fractions, sections were incubated in 3% hydrogen peroxide to prevent endogenous peroxidase activity. 

The sections that were treated with anti-Ob primary antibody (leptin) (sc-842), anti- galanin (sc-5446) and anti-GLUT2 (sc-9117) were blocked with Ultra V Block in the ready-to-use biotinylated goat anti-polyvalent kit (TP-125-BN) for 10 min at room temperature. Subsequently, primary antibodies were incubated overnight at +4 ^°^C. After washing 4 times with PBS, biotinylated goat anti-polyvalent was incubated for 15 min at room temperature. After sections were washed 4 times with PBS, enzyme-labeled streptavidin was applied. Afterwards, aminoethyl carbazole (AEC) chromogen was applied. The reaction waited until it was observed. The reaction was stopped with tap water. After the ground staining with hematoxylin was performed, it was closed with water based sealer. The sections were imaged with the Olympus BX-51 brand light microscope.

***Tissue preparation for Western blot***

Expressions of leptin and GLUT2 were measured by western blot in liver tissues, which were cut into small pieces and lysis buffer was added and waited for 5 min at room temperature. The samples were then homogenized and centrifuged at 14000 g for 10 min at 4 ^°^C. The supernatant that included the cytosolic proteins was transfered to a new tube. The protein concentration was then measured with the nanodrope by means of a fluorometer (Qubit 2.0 Fluorometer, Invitrogen, Thermo Fisher Scientific, Waltham, USA). 30 μg protein was put in 10% SDS gel for electrophoresis, then transferred to PVDF membrane. Membranes were blocked with blocking buffer 5% BSA (bovine serum albumin) for 1 hr at room temperature. The membranes were then incubated with appropriate anti-Ob (sc-842), anti-GLUT2 (sc-9117) and anti- β-actin (sc-47778) antibodies overnight at 4 ^°^C. Membranes were washed 3 times with the washing solution for 10 min, then incubated with appropriate secondary antibody (sc- 2004). Protein expression levels were observed in immunoreactive strips by imaging system (C-Digit, Licor, Cambridge, United Kingdom). Analysis of image results were performed with Image J 1.49v.

***Statistical analysis***

Blood glucose measurement data were analyzed using a commercially available software Statistics Package for Social Sciences (SPSS for Windows, version 18.0, Chicago, Illinois, USA). Results were expressed as mean±SEM. *P*-values less than 0.05 were considered to be significant. All of the data were tested for a normal distribution with the Shapiro-Wilk test to determine whether the results should be analyzed parametrically. All of the experimental data displayed normal distributions, and we therefore used parametric tests. For the comparisons of more than two groups, we used the one-way analysis of variance. We used Tukey’s honestly significant difference (HSD) test for multiple comparisons.

## Results

***Mirtazapine decreased blood glucose level***

**Figure 1:**
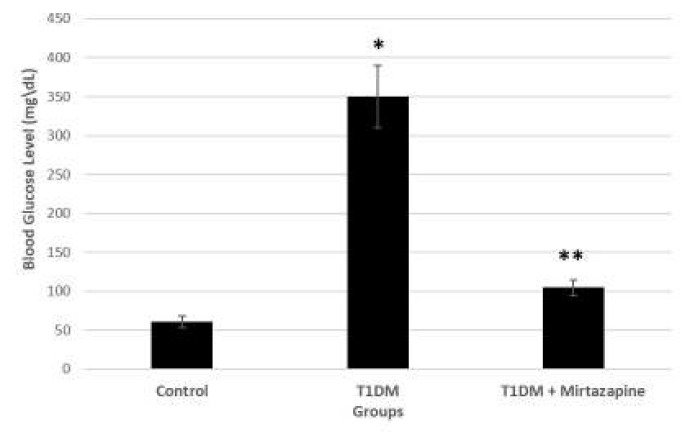
Effect of intragastric mirtazapine applied on diabetic rats

STZ treatment resulted in more than threefold increase in blood glucose level. Glucose level varied from 308 to 402 mg/dl. Administration of mirtazapine to diabetic rats (T1DM) for 2 weeks reduced blood glucose level and shows significant differences in blood glucose level as compared to diabetic group, and there was also significant differences in blood glucose level between diabetic and diabetic+mirtazapine groups (T1DM+Mirtazapine) (Figure 1). 

***Mirtazapine increased stored glycogen in liver***

**Figure 2:**
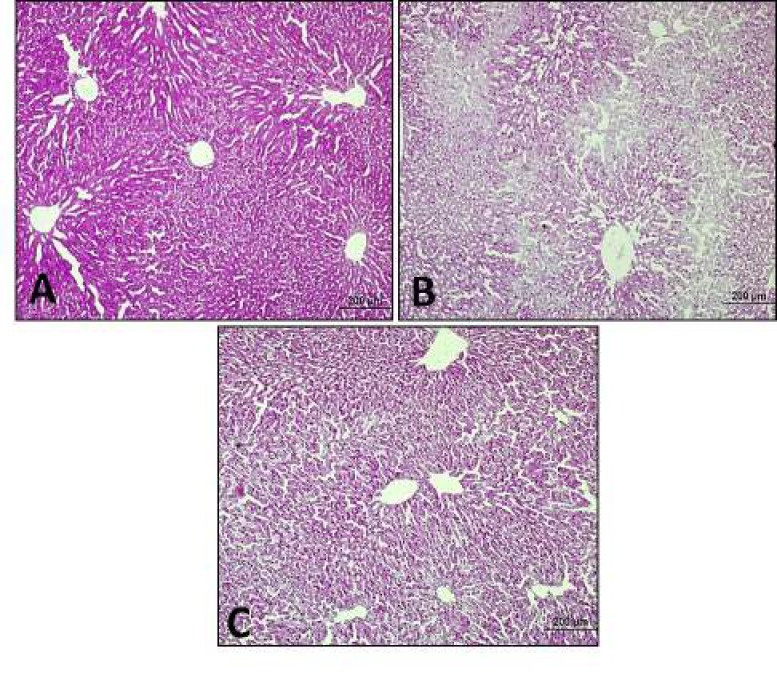
Periodic Acid Schiff (PAS) reactions

According to the results of our study, there were healthy hepatocytes having radial structure and hepatocytes containing stored glycogen in PAS reaction in the control group, whereas glycogen amount decreased in diabetic group. After 20 mg/kg mirtazapine administration to diabetic rats, increased glycogen storage were observed (Figure 2).

***Mirtazapine increased leptin reaction reduced by diabetes***

**Figure 3:**
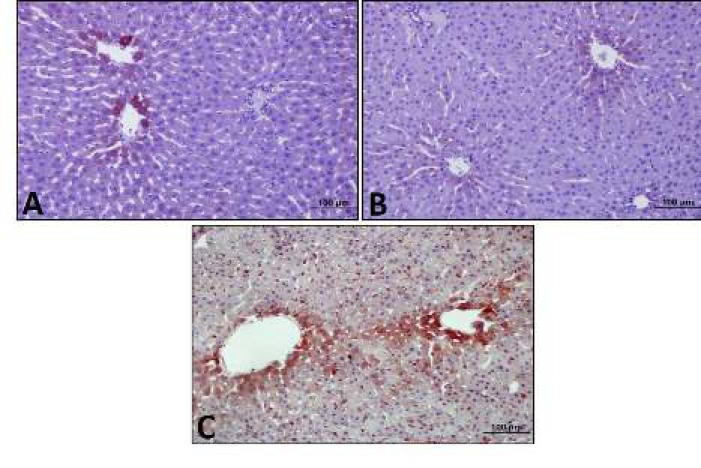
Leptin expression by immunohistochemistry

According to the results of the immunohistochemistry, middle leptin expression was observed in control group. Low leptin expression was observed in diabetes group as control group, whereas intensive leptin expression was observed in mirtazapine-applied diabetes goup especially near vena centralis (Figure 3).

**Figure 4:**
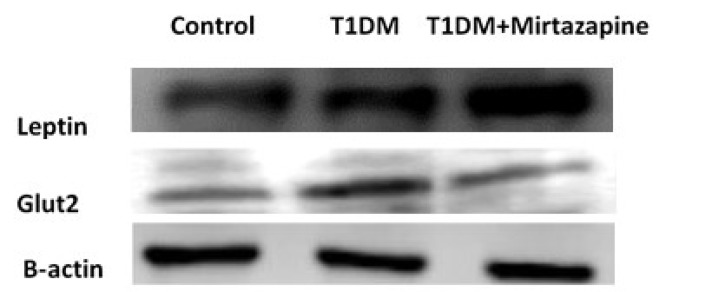
Leptin and galanin expression by Western blot

**Figure 5:**
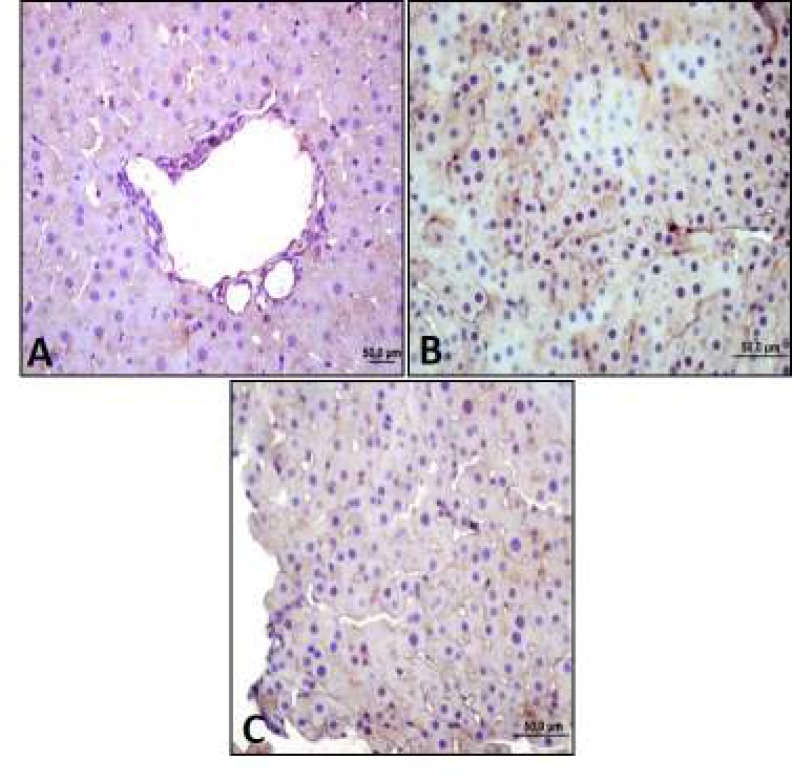
Leptin and Glut2 expression measurements by Western blot

When we observed leptin expressions by western blot, we saw that leptin expression decreased 1.14 fold in diabetes group and increased 1.29 fold in mirtazapine-administered diabetes group compared to control group. It was increased 0.36 fold in mirtazapine-administered diabetes group compared to diabetes group (Figures 4-5).

Streptozocin (STZ) injection was performed on day 0 and mirtazapine was applied daily starting on the 4th week till 6th week. All of the data were tested for a normal distribution with the Shapiro-Wilk test to determine whether the results should be analyzed parametrically. For the comparisons of more than two groups, we used the one-way analysis of variance. We used Tukey’s honestly significant difference (HSD) test for multiple comparisons. Significant difference between control and diabetes group * *P<*0.05 and significant difference between diabetes and diabetes+mirtazapine groups ** *P<*0.05

Intensive PAS (+) reaction in hepatocytes in control group (A). Decreased PAS (+) reactions in diabetic group (B). Increased PAS (+) reactions in hepatocytes in mirtazapine administered to diabetic group (C) (10x)

Middle anti-leptin antibody reaction in control group (A). Low anti-leptin antibody reaction in diabetes group (B). Intensive anti-leptin antibody reaction especially near vena centarlis in mirtazapine administered to diabetes group (C) (20x)

**Figure 6:**
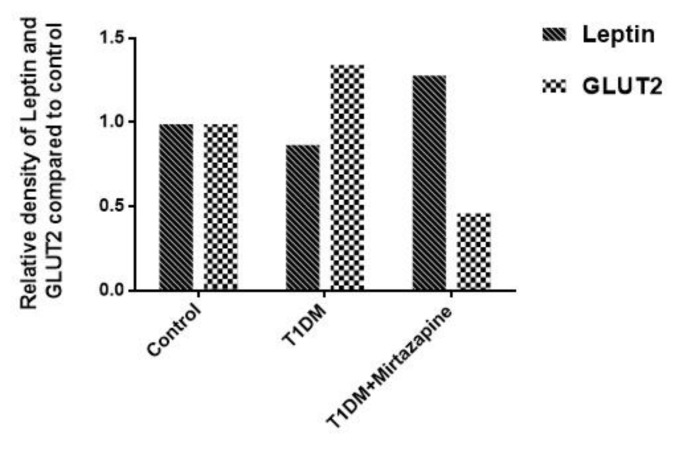
Glucose transporter 2 (GLUT2) expression by immunohistochemistry

Middle anti-GLUT2 antibody reaction in control group (A). Intensive anti-GLUT2 antibody reaction in diabetes group (B). Low anti-GLUT2 antibody reaction in mirtazapine administered to diabetes group (C) (40x)

**Figure 7:**
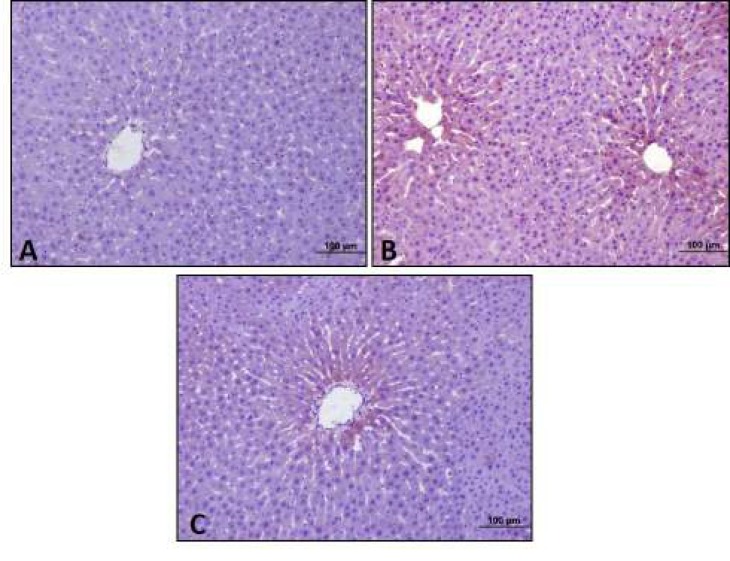
Galanin expression by immunohistochemistry

Low anti-galanin antibody reaction in control group (A). Intensive anti-galanin antibody reaction in diabetes group (B). Middle anti-galanin antibody reaction in mirtazapine administered to diabetes group (C) (20x)

***Mirtazapine decreased GLUT2 and galanin reactions increased by diabetes***

According to the results of immunohistochemistry and western blot analysis in our study, GLUT2 expressions increased in type 1 diabetes group when compared to control group. However, we observed that GLUT2 expressions decreased in mirtazapine-administered diabetes group. Analysis of GLUT2 expressions by western blot showed that GLUT2 expression increased 1.35 fold in diabetes group and decreased 0.47 fold in mirtazapine-administered diabetes group compared to control group (Figures 4-6). Despite the increased expression of GLUT2, T1DM animals presented a significant reduction in the amount of glycogen when compared to control animals.

Also, investigation of galanin expressions by immunohistochemistry analysis showed that galanin expressions increased in diabetes group when compared to control group. However, after mirtazapine administration to dibatic rats, we saw that galanin expressions decreased (Figure 7).

## Discussion

This study was planned to investigate possible anti-hyperglycemic activity of mirtazapine. Further, promising contribution of leptin, galanin and GLUT2 channel to the pharmacological activity of mirtazapine were elucidated by conducting moleculer methods. 

T1DM is a disease characterized by insulin deficiency due to the autoimmune destruction of pancreatic beta cells. It becomes manifest when the remaining beta cell mass is not able to secrete sufficient amounts of insulin required for the maintenance of normal glucose homeostasis. Adipocytokines are hormones secreted by the adipose tissue. Adipocytokines’ main role is signalling key organs to maintain metabolic homeostasis, and their dysfunction has been causally linked to a wide range of metabolic diseases. Adipocytokines are thoroughly explored in obesity and related metabolic disorders, but there is much less data concerning serum levels of different adipocytokines in T1DM patients, especially in the context of presence of different chronic complications of T1DM. While leptin is a target of many trials in T2DM patients, the number of studies assessing the significance of leptin in T1DM is modest. Various authors reported different results regarding the serum leptin levels in T1DM in contrast to nondiabetic individuals. While some have come to the conclusion that the serum leptin levels have been increased, others have reported decreased levels and the third ones have concluded that the levels have been unchanged (20). 

Diabetes mellitus and depression are two chronic and prevalent diseases, which affect millions of people all over the world. Galanin, a 29-amino acid peptide hormone, is widely distributed throughout the central and peripheral nervous systems as well as other tissues (21) to modulate depression (22-24), cognition, neuroendocrine, Alzheimer’s disease (25), neuronal differentiation (26), pain threshold (27), food intake and energy homeostasis (28). Although galanin may directly inhibit β cell secretion via G(o)2 of the G(i/o) protein family, the inhibitive effect of galanin on insulin secretion does not interfere with its beneficial role to insulin sensitivity of patients. The plasma galanin level in patients with diabetes is higher than in healthy controls, resulting from impaired glucose clearance activity (29). Also, galanin attenuates depression-like behavior and insulin resistance through the central galanin receptor 2 (GalR2) (30). 

The glucose transporter isoform GLUT2 takes part in essential roles in the complex pathways mediating entire body glucose disposal, in which dysregulation of their controlling mechanism can result in the pathophysiologic conditions related to diabetes (31). GLUT2, the insulin independent main hepatic glucose transporter, has been shown to be overexpressed in T1DM animals. This is in agreement with several studies, reporting that hyperglycemia and several nutritional factors regulate the expression of GLUT2 in liver and kidney (32).

Leptin, insulin and nutrient-related signals in the central nervous system (CNS) improve hepatic insulin sensitivity; however, the mechanism by which CNS leptin signaling normalizes diabetic hyperglycemia and whether this process involves leptin-dependent effects on hepatic glucose production or tissue glucose uptake are unknown. Leptin action in the brain potently suppresses hepatic glucose production, while increasing tissue glucose uptake despite persistent and severe insulin deficiency (33). 

A study by Schilling and colleagues showed that antidepressant treatment with venlafaxine and paroxetine does not impact on leptin plasma concentrations, whereas treatment with amitriptyline and mirtazapine leads to an increase of leptin secretion even when controlling for body mass index (BMI) (4). Mirtazapine is strong antagonist at the histamine H1-receptor, and is known to be involved in the regulation of body weight. Blockade of histamine synthesis as well as blockade of H1-receptors has been shown to attenuate the response to leptin (34).

Schmid and collegues showed that leptin concentrations increased after 4 weeks of treatment depressive disorder patients with mirtazapine (35).

The regulation of beta cell function in the short and long term allows production of an adequate level of plasma insulin levels, which restores plasma glucose concentrations to normoglycemia by inducing glucose uptake and accumulation as glycogen and fatty acids, principally in muscle, liver, and adipose tissue. However, a decrease in beta cell mass or impaired beta cell function can lead to abnormal plasma insulin levels that can promote glucose intolerance and diabetes (36). It has been implicated that alpha-adrenergic antagonists play role on storage of glycogen by reducing the glucose concentration in the plasma without altering the insulin response (37). Mirtazapine inhibits α2 adrenergic auto and heteroreceptors, and in our study we showed that the amount of glycogen storage was significantly increased in the liver, and blood glucose level decreased in mirtazapine-treated diabetic rats.

Alone, leptin has been reported to promote glycogen storage in hepatocytes by inhibiting glycogen phosphorylase and glycogen synthase kinase 3 (GSK3) in perfused rat liver and hepatic cell lines, and to inhibit glucose production in response to gluconeogenic precursors in the perfused rat liver and isolated hepatocytes (38). Leptin can also inhibit glucagon action in primary rat hepatocytes and the perfused rat liver (39), an effect that is possibly mediated through activation of phosphoinositide 3-kinase (PI3K) and phosphodiesterase 3B (PDE3B) (40). Collectively, these studies show that leptin directly modulates insulin signaling and glucose flux, but the effects of leptin are highly variable. Perhaps contributing to these seemingly contradictory findings is that the effect of leptin on hepatocytes appears to be dependent on duration of leptin pretreatment (41), species (42) and nutritional status (43). Indeed, one study found that perfusion of livers during the postprandial state inhibited epinephrine-stimulated glucose production, whereas perfusion of livers during the postabsorptive phase stimulated glucose release (44). 

Perhaps the most compelling evidence of the profound effect of leptin on glucose homeostasis is that leptin administration can normalize blood glucose levels in non-obese rodent models of insulin deficient, T1DM. Leptin infusion or gene therapy, can reverse hyperglycemia without a detectable rise in circulating insulin levels in STZ-treated rats (33) and mice (45), non-obese diabetic (NOD) mice (46), insulin deficient Akita mice (46) and BioBreeding rats with virally-induced beta cell destruction. Parallel to all these studies, in our study, leptin level increased, GLUT2 and galanin levels decreased after mirtazapine administration, and it caused an increase in glucogen storage as well as mirtazapine’s inhibitor effect on α2 adrenergic auto and heteroreceptors.

These results further support evidence that mirtazapine ameliorates hyperglycemia by decreasing GLUT2 through leptin and galanin in the liver of type 1 diabetic rats. Mirtazapine can be used for both type 1 diabetes-induced depressive mood as an antidepressant and for decreased blood glucose level. 

## Conclusion

This data show, for the first time, that mirtazapine has beneficial effects against diabetes-induced hyperglycemia, and that suppressive effect of this drug on GLUT2 and leptin, and upregulation effect on galanin levels, which are altered in diabetic rats, may be some of the pharmacological mechanisms underlying the exhibited anti-hyperglycemic effect of mirtazapine.
